# Nonparametric bootstrap methods for interval estimation of the area under the ROC curve with correlated diagnostic test data: application to whole-virus ELISA testing in swine

**DOI:** 10.3389/fvets.2023.1274786

**Published:** 2023-12-05

**Authors:** Jinji Pang, Wangqian Ju, Michael Welch, Phillip Gauger, Peng Liu, Qijing Zhang, Chong Wang

**Affiliations:** ^1^Department of Statistics, Iowa State University, Ames, IA, United States; ^2^Department of Veterinary Microbiology and Preventive Medicine, Iowa State University, Ames, IA, United States; ^3^Department of Veterinary Diagnostic and Production Animal Medicine, Iowa State University, Ames, IA, United States

**Keywords:** receiver operating characteristic (ROC) curve, area under the curve (AUC), correlated data analysis, diagnostic test, cluster bootstrapping, hierarchical bootstrapping

## Abstract

Developing and evaluating novel diagnostic assays are crucial components of contemporary diagnostic research. The receiver operating characteristic (ROC) curve and the area under the ROC curve (AUC) are frequently used to evaluate diagnostic assays’ performance. The variation in AUC estimation can be quantified nonparametrically using resampling methods, such as bootstrapping, and then used to construct interval estimation for the AUC. When multiple observations are observed from the same subject, which is very common in veterinary diagnostic tests evaluation experiments, a traditional bootstrap-based method can fail to provide valid interval estimations of AUC. In particular, the traditional method does not account for the correlation among data observations and could result in interval estimation that fails to cover the true AUC adequately at the desired confidence level. In this paper, we proposed two novel methods to calculate the confidence interval of the AUC for correlated diagnostic test data based on cluster bootstrapping and hierarchical bootstrapping, respectively. Our simulation studies showed that both proposed methods had adequate coverage probabilities which were higher than the existing traditional method when there were intra-subject correlations. We also discussed applying the proposed methods to evaluate a novel whole-virus ELISA (wv-ELISA) diagnostic assay in detecting porcine parainfluenza virus type-1 antibodies in swine serum.

## 1 Introduction

In pursuit of the One Health initiative, which aims to safeguard the health and well-being of humans and animals, researchers have made substantial efforts to develop new diagnostic tests through animal and human studies. A receiver operating characteristic curve, or an ROC curve, is a useful graphical tool to visualize, assess, select, and compare tests in diagnostic evaluation and decision-making (1–4). The ROC curve is obtained by plotting sensitivity against 1-specificity at various threshold settings, where sensitivity is also known as the true positive rate and specificity the true negative rate (1). The area under the curve (AUC) is widely used as an index associated with an ROC curve. In practice, many experiments examining the efficiency of new diagnostic tests, especially veterinary experiments, involve taking multiple observations from the same subjects, resulting in correlated data structures (5–8). A traditional bootstrap-based method may fail to provide valid interval estimations of AUC. In particular, the traditional method does not account for the correlation among data observations and would result in interval estimation that does not cover the true AUC at the desired confidence level. Due to the popularity of such experiments with repeated measures design, there is an urgent need to develop new methodologies to calculate the confidence interval of the AUC for correlated data with a legitimate coverage level.

For a diagnostic test with more than two possible test outcomes, a cutoff value can be selected to dichotomize the original test outcome into a binary positive or negative result. As the cutoff value varies through all possible choices, the sensitivity and specificity change accordingly, thus forming the ROC curve. By plotting the ROC curve, researchers can visually compare the tradeoff between the sensitivity and the specificity of the corresponding diagnostic test and determine the effectiveness of the test. ROC analysis has been extensively used in continuous and ordinal scaled diagnostic test evaluation (4, 9). The AUC can be interpreted as the probability that a random abnormal subject has a diagnostic test outcome that is worse than a random normal subject. Empirical estimation of the AUC can be calculated by the trapezoidal rule for an empirical ROC curve, which is also known as the concordance C-statistic and related to the Wilcoxon-Mann–Whitney U-Statistic (3). The ROC curve and its AUC do not depend on specification of a cutoff value and can be considered as comprehensive measures of the discriminating ability of a non-binary diagnostic test.

To construct interval estimation of the AUC, e.g., a 95% confidence interval (CI) of AUC, a nonparametric bootstrap method is frequently used. The nonparametric bootstrap method constructs bootstrap samples by sampling with replacement from the original dataset and was first introduced by Bradley Efron (10). Unlike the conventional asymptotic approach based on large sample sizes, the bootstrap method is effective in its lack of assumptions regarding the underlying data distribution. A traditional bootstrap method resamples observations independently with replacement from the original data, without consideration of any correlation structures. For example, the pROC package, currently one of the most frequently used tools for analyzing ROC curves, can perform confidence interval estimation with such traditional bootstrap methods (11). Since its first release on BMC Bioinformatics in March 2011, the pROC package has been cited over 6,500 times (11). However, the traditional nonparametric bootstrap method ignores potential correlation structure in data and thus can fail to produce legitimate interval estimation when multiple observations are taken on the same subject.

Various bootstrap methods have been proposed for datasets with complicated structures, including correlated data. Field and Welsh discussed the topic of bootstrapping clustered data in 2006 (12). A cluster bootstrap method is performed by bootstrapping clusters using simple random resampling with replacement without changing the observations within the selected clusters. This is a simplified version of the randomized cluster bootstrap discussed by Davison et al. (13). Alternatively, a hierarchical bootstrap method creates new bootstrap datasets by resampling with replacement at multiple levels, usually from the highest level down to the lower level. Hierarchical bootstrap has been applied successfully to multi-level datasets and outperforms the traditional statistical tests (14).

Although bootstrap methods have been developed for clustered or hierarchical data structures, these bootstrap methods have not been applied to ROC analysis for correlated diagnostic test data. Besides nonparametric methods, the correlation among repeated measurements from the same subjects can be alternatively modeled using mixed models with subjects as a random effect. Liu et al. proposed to test statistical significance related to the AUC of an ROC curve under a repeated measures design through a generalized linear mixed model (GLMM) (15). The package of Liu et al. is yet under development; thus, we cannot implement the method (through communication with the author). There is a need to develop new methodologies and tools to calculate the confidence interval of the AUC for correlated data with a legitimate coverage level and make them available. Here we focus on developing nonparametric methods based on the concept of bootstrapping (resampling).

In this paper, we considered the correlation structure caused by the clusters in ROC analysis and propose to utilize the cluster bootstrap and the hierarchical bootstrap methods to achieve legitimate interval estimation of the AUC. We evaluated the impact of this correlation on the ROC analysis by comparing three nonparametric bootstrap methods:

1. Traditional bootstrap method.
2. Cluster bootstrap method.
3. Hierarchical bootstrap method.

Simulation studies were conducted to compare the performances of the three methods in terms of coverage probability and 95% CI width of AUC. A dataset from a real diagnostic study that developed and compared three assays to detect porcine parainfluenza virus type-1 antibody in swine serum was used to demonstrate the application of the proposed methods.

## 2 Materials and methods

### 2.1 Sample notations and assumptions

In diagnostic trials, it is frequently observed that multiple observations are often collected from each of the many subjects. Let *Y_ij_* denotes the *j^th^* observation from the *i^th^* subject, where *i* = 1, 2, 3, …, *K*, *j* = 1, 2, 3, …, *n_i_.* In total, there are *N* = $\overset{K}{\underset{i=1}{\sum }}n_{i}$ observations from *K* subjects.

### 2.2 Traditional bootstrap method

The traditional bootstrap method resamples at the observation level and is the method implemented in the pROC package. Traditional bootstrap ignores the correlation of observations from the same subject. This method would be appropriate if we can assume *N* observations from *K* subjects are independent of each other, though multiple observations could come from the same subject in our example scenario. Nonparametric bootstrap will be performed on *N* individual observations by simply resampling with replacement. Each time, the same number of observations will be selected, which is *N*.

The nonparametric bootstrap procedure is:

1. Assume we have a vector ***x*** = *(x_1_, …, x_N_)*^T^ of length *N* to represent all the observations in the original sample of data. Sampling with replacement involves forming a new vector ***x***^***∗***^ = (x_1_*,…, x_N_*)^T^, where each x_i_* is independently sampled from ***x*** with equal probability given to each observation, i.e., P (x_i_* = *x_j_*) = 1/*N* for all *i* and *j*.
2. The bootstrap sample of a replicate will be all the observations sampled.
3. One thousand bootstrap samples will be generated, and the 95% CI for AUC will be calculated based on the bootstrap samples.

### 2.3 Cluster bootstrap method

The cluster bootstrap method considers the correlation of observations from the same subject (cluster). It assumes observations from the same subject are correlated. For each resampled data set, *K* subjects will be sampled from the original subject pool with replacement. Then, all the observations from the K subjects will be sampled to form the bootstrap data. The number of observations of each bootstrap sample may vary, as different subjects can have different numbers of observations.

The cluster bootstrap procedure is:

1. We have vector ***k*** *= (k_1_, …, k_K_)*^T^ of length *K* to represent all the unique subjects in the original sample of data. Sampling on subjects with replacement involves forming a new vector k* = (k_1_^*^,…, k_K_*)^T^, where each k_i_* is independently sampled from ***k*** with equal probability given to each subject, i.e., P (k_i_* = k_j_) = 1/*K* for all *i* and *j.*
2. For each sampled subject k_i_*, where k_i_* = k_j_, all the observations will be sampled (without randomness) from this subject *k_j_*.
3. A bootstrap sample of data contains all such observations from all sampled subjects k* = (k_1_*,…, k_K_*)^T^.
4. One thousand bootstrap samples will be generated, and the 95% CI for AUC will be calculated based on the bootstrap samples.

### 2.4 Hierarchical bootstrap method

Similar to the cluster bootstrap method, the hierarchical bootstrap method also considers the correlation of observations from each subject. Nonparametric bootstrap will be performed on *K* subjects first. Each time, *K* subjects will be selected from the original subject pool with replacement, which is the same as in a cluster bootstrap. Then, observations will be selected from a subject with replacement, with the number of observations per subject to be the same as in the original data for this subject. This step differs from the cluster bootstrap, where observations within a subject are not resampled. The bootstrap sample will contain all the sampled observations from all the sampled subjects. Similar to cluster bootstrap, the number of observations of each bootstrap sample may vary, as different subjects can have different numbers of observations.

The hierarchical bootstrap procedure is:

1. Let vector ***k*** = (*k_1_*, …, *k_K_*)^T^ of length *K* represent all the unique subjects in the original sample of data. Resampling on subjects with replacement involves forming a new vector k* = (k_1_*,…, k_K_*)^T^, where each ki* is independently sampled from ***k*** with equal probability given to each subject, i.e., P (k_i_* = *k_j_*) = 1/*K* for all *i* and *j*. Note that this step is the same as step 1 of the cluster bootstrap method.
2. For each ki* from k*, where k_i_* = *k_j_*, *n_j_* observations will be sampled from subject *k_j_* with replacement with equal probability.
3. One hierarchical bootstrap sample of data will be all the sampled observations from all the sampled subjects. Empirical ROC is calculated according to the bootstrap sampled data and the corresponding AUC is calculated nonparametrically.
4. One thousand bootstrap samples will be generated, and the 95% CI for AUC will be calculated based on the bootstrap samples.

### 2.5 Application to a novel whole-virus ELISA diagnostic assay

The dataset is from a published paper that utilizes different diagnostic assays to detect porcine parainfluenza virus type-1 antibody in swine serum (16). The dataset has 364 observations in total, including 168 porcine parainfluenza virus 1 (PPIV-1) unchallenged observations and 196 PPIV-1 challenged observations from 72 unique subjects. The animals in the challenged group were inoculated intratracheally and intranasally with tissue culture isolate, and the challenge statuses were confirmed by the RT-rtPCR test for all observations. Approximately 70% of the subjects have 6 observations each, and the remaining 30% have 1 to 5 observations each. For subjects with 6 observations each, 40% of the subjects only have unchallenged observations, which means the challenge status of the 6 observations are all unchallenged, while 60% of the subjects have different challenge statuses as the first observation is unchallenged and the following 5 observations are challenged. Antibodies of the serum samples were measured by wv-ELISA as described previously (16). Our proposed methods based on the cluster bootstrap and the hierarchal bootstrap are applied to analyze this diagnostic test data and compared to the traditional bootstrap method. Results are presented and discussed in the sections below.

### 2.6 Validation with simulation studies

To validate and check the robustness of our methods, we conducted two simulation studies. The validity of the proposed methods as well as the traditional method were evaluated using the coverage probabilities of the 95% confidence interval of AUC. The coverage probability is the probability that the calculated 95% confidence interval covers the true AUC value. A legitimate 95% confidence interval is expected to cover the true value of interest 95% of the time as the study is repeated randomly. A coverage probability much lower than the desired level is of severe concern as there is a high chance that the interval misses the true value. The coverage probability can be calculated as the proportion that the calculated confidence interval contains the true AUC value out of all simulated data sets.

The simulation parameters were estimated from the above ELISA application data by fitting the dataset to a linear mixed effect model:


(1) $$Y_{ij}=\beta_{0}+\beta_{1}\times S_{ij}+\tau_{i}+\epsilon_{ij}$$

Here $Y_{ij}$ represents the outcome value of the $j^{th}$ observation from the $i^{th}$ subject; $\beta_{0}$is the mean value of the response variable when the predictor variable is 0; $\beta_{1}$is the mean difference between the challenged status and the unchallenged status; $S_{ij}$ is the indicator function denotes the challenge status of the $j^{th}$ observation from the $i^{th}$ subject, where $S_{ij}=1$ when the challenge status is challenged and $S_{ij}=0$ otherwise; $\tau_{i}\overset{iid}{\sim }N\left(0,\sigma_{s}^{2}\right)$ is the random effect of the subject *i*, and $\epsilon_{ij}\overset{iid}{\sim }N\left(0,\sigma_{e}^{2}\right)$ is the random observational level error term.

By analyzing the real data, the unchallenged population’s wv-ELISA distribution appears to follow a normal distribution, $N\left(\beta_{0},\sigma^{2}\right)\;\text{.}$ In comparison, the wv-ELISA distribution of the challenged population follows a normal distribution with a different mean, $N\left(\;\beta_{0}+\beta_{1},\sigma^{2}\right)$. Here $\sigma^{2}=\sigma_{s}^{2}+\sigma_{e}^{2}$ is the total variation. According to the fitted linear mixed effect model, we estimated the coefficients from the analysis of the real data:


$$\left\{ \begin{array}{c} \sigma_{s}^{2}=0.2381\\ \sigma_{e}^{2}=0.2936\\ \sigma^{2}=0.5317 \end{array}\right.$$

Two simulation studies were performed to assess and compare the proposed methods to the traditional bootstrap method. In the first simulation study, the data were simulated to examine a situation where the challenge status of observations from the same subject may change over time, mimicking the structure of the real data. For each simulated dataset, 100 subjects were simulated, with 6 observations from each of the subjects. Among these subjects, P_1_ (=40) subjects were assigned to the unchallenged group, where the challenge status of all the observations from these subjects was always unchallenged. The other P2 (=60) subjects were assigned to the challenged group, where out of the 6 observations for each subject, the first observation had a status of unchallenged, and the remaining 5 observations were challenged.

A second simulation study was performed to examine a situation where the challenge status of observations from the same subject does not change over time. The data structure is similar to that of the first study except that the statuses of all 6 observations of the P_2_ challenged subjects were fixed to be challenged all the time.

The parameters for the two simulation studies were chosen based on the estimates from the real data analysis. Six combinations of the simulation parameters were considered in each simulation study, namely $\beta_{0}=-0.0564$;$\beta_{1}$= 1.3873 or 0.6936; {$\sigma_{s}^{2},\sigma_{e}^{2}\}$= {0.2381, 0.2936}, {0.01, 0.5217}, or {0.5217, 0.01}. For the mean parameters, we considered the estimated effect size 1.38728, and half of that, 0.6936. The variance parameters were set up so that we controlled each combination’s total variance ($\sigma_{s}^{2}+\sigma_{e}^{2})$ to be equal. Two thousand datasets were simulated and analyzed for each parameter combination using the three bootstrap methods discussed above.

## 3 Results

### 3.1 Real data analysis

**Figure 1:**
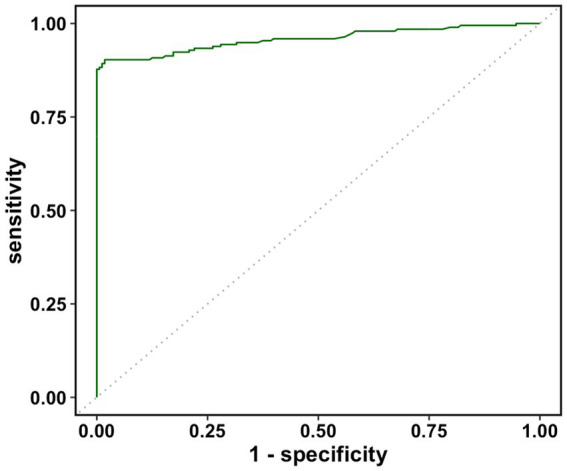
Estimated receiver operating characteristic (ROC) curve of wv-ELISA diagnostic assay.

The real data was analyzed by the three methods discussed previously. The wv-ELISA test result was a continuous random variable, and the challenge status was binary, either positive or negative. The range for wv-ELISA results was from −0.160 to 3.515. We chose different thresholds between −0.160 to 3.515 to calculate the sensitivity and (1-specificity) and plot the sensitivity against the (1-specificity) to plot the ROC curve. The point estimate was determined by a specific threshold value and the corresponding sensitivity and (1-specificity). As seen in Figure 1, the estimated ROC curve of wv-ELISA bulged to the upper left point (0,1) and the estimated AUCs were high. The mean, the 95% CI, and the width of the AUC were calculated by the three methods and summarized in Table 1. As we can see from Table 1, the three methods gave similar mean estimates of the AUC. The 95% CI widths of AUC differed between each method, with the cluster and traditional bootstrap method having similar widths, whereas the hierarchical bootstrap method had the widest width. The large value of AUC implies that wv-ELISA is a highly effective tool in detecting porcine parainfluenza virus type-1 antibody.

**Table 1:** AUC and 95% confidence interval were calculated by three bootstrap methods.

Method	AUC	95% Confidence interval		
		Lower bound	Upper bound	Width
Traditional	0.958	0.9355	0.9776	0.0421
Cluster	0.9583	0.9352	0.9757	0.0405
Hierarchical	0.9586	0.9283	0.9814	0.0531

### 3.2 Simulation studies results

Coverage probabilities and 95% confidence interval widths of AUC were evaluated for the three methods using the simulated datasets. The coverage probability is the probability that the calculated 95% confidence interval covers the true AUC, which is 0.910741 or 0.7494095, depending on the mean difference parameter ($\beta_{1}$). The 95% confidence interval width of AUC is the difference between the upper bound and the lower bound of the calculated 95% confidence interval. A legitimate 95% confidence interval is expected to cover the true parameter of interest 95% of the time as the study is repeated randomly. A coverage probability much lower than the desired level is of severe concern as there is a high chance that the interval misses the true parameter value.

The results of simulation studies 1 and 2 were summarized in Tables 2, 3, respectively. Table 2 shows that when the subject’s random effect was big, the traditional bootstrap method gave the lowest coverage probability. In particular, the coverage probability for traditional method ranged from 62.75% to 82.05% for settings 1,2,5, and 6 in simulation study 1 (Table 2). This low coverage probability is due to the empirical method’s neglect of the correlations between observations from the same subject, and therefore it is invalid to use when the random effect of the subject is big. However, when the random effect of the subject was extremely small, the traditional bootstrap method is legitimate since the coverage probabilities were close to 95%. The proposed cluster bootstrap and hierarchical bootstrap methods gave high coverage probabilities regardless of the random effect of the subject. The hierarchical bootstrap method always gave higher coverage probabilities than the cluster bootstrap method; however, we should also note that the 95% CI widths of AUC of the hierarchical bootstrap method were also wider than the cluster bootstrap method.

**Table 2:** Simulation study 1: the challenge status of observations from the same subject may change over time.

	Simulation parameters			Traditional		Cluster		Hierarchical	
No.	$\beta_{1}$	$\sigma_{s}^{2}$	$\sigma_{e}^{2}$	Coverage	Width	Coverage	Width	Coverage	Width
1	1.38728	0.2381	0.2936	82.05%	0.0444	93.75%	0.0649	96.25%	0.0742
2	0.69364	0.2381	0.2936	81.0%	0.0767	94.3%	0.1149	97.05%	0.1299
3	1.38728	0.01	0.5217	93.55%	0.0446	93.75%	0.0451	99.15%	0.0609
4	0.69364	0.01	0.5217	94.95%	0.0768	94.65%	0.0779	98.95%	0.1047
5	1.38728	0.5217	0.01	62.75%	0.0443	93.75%	0.0938	94.85%	0.0966
6	0.69364	0.5217	0.01	64.65%	0.0766	94.05%	0.1572	94.7%	0.1632

Coverage probabilities and confidence interval widths were calculated by 2000 simulated datasets under 6 simulation parameter settings. True AUC for No. 1, No. 3, No. 5 is 0.910741, for No. 2, No. 4, No. 6 is 0.7494095.

**Table 3:** Simulation study 2: the challenge status of observations from the same subject does not change over time.

	Simulation parameters			Traditional		Cluster		Hierarchical	
No.	$\beta_{1}$	$\sigma_{s}^{2}$	$\sigma_{e}^{2}$	Coverage	Width	Coverage	Width	Coverage	Width
1	1.38728	0.2381	0.2936	75.05%	0.0452	93.7%	0.075	95.15%	0.0822
2	0.69364	0.2381	0.2916	74.3%	0.078	94.55%	0.1361	96.0%	0.147
3	1.38728	0.01	0.5217	93.6%	0.0454	94.2%	0.0465	98.9%	0.0625
4	0.69364	0.01	0.5217	94.25%	0.0782	94.7%	0.0808	99%	0.1079
5	1.38728	0.5217	0.01	55.2%	0.0449	94.25%	0.1095	94.55%	0.1098
6	0.69364	0.5217	0.01	57.1%	0.0782	94.2%	0.1935	94.15%	0.194

Coverage probabilities and confidence interval widths were calculated by 2000 simulated datasets under 6 simulation parameter settings. True AUC for No. 1, No. 3, No. 5 is 0.910741, for No. 2, No. 4, No. 6 is 0.7494095.

From Table 3, the results of simulation study 2 were very similar to those of simulation study 1. The cluster bootstrap method and the hierarchical bootstrap method gave high coverage probabilities regardless of the random effect of the subject as before, and the traditional bootstrap method only gave rational coverage probability when the subject’s random effect was insignificant. However, two results differed from simulation study 1. First, the traditional bootstrap method had an approximate 7% drop in coverage probabilities when the subject’s random effect was noteworthy. Second, there was no significant difference between the cluster bootstrap and the hierarchical bootstrap methods when the subject’s random effect was big. Those two differences in results can be explained as the subject’s random effect from the same subject will only affect the unchallenged or challenged populations, which increases the effect caused by it while implementing ROC analysis. In contrast, in simulation study 1, the subject’s random effect of the challenged group affected both the challenged and unchallenged populations in ROC analysis.

## 4 Discussion

The area under the ROC curve is a widely used tool to measure the accuracy of diagnostic tests, and there are many examples where it has been applied extensively in veterinary diagnostic test studies (17–20). However, when repeated measurements from the same subject have been collected, the correlation of observations from the same subject is always ignored in the statistical analysis (5–8). Furthermore, although it occurred in only a small number of experiments, the challenge status of a subject can also change if repeated measurements are collected longitudinally. The weighted area under the ROC curve method has been proposed to solve multi-reader multi-test data in medical imaging modality studies (21). Michael et al. proposed a model where the biomarker levels were conditioned on the previous status of a patient to perform ROC analysis for regularly measured biomarkers longitudinally (22). However, neither of these two methods fits into the scenario which often occurs in veterinary diagnostic tests. Therefore, the lack of proper methods and the need to analyze correlated data in veterinary medicine research motivated us to develop methods that can be used in veterinary diagnostic studies where multiple observations are always drawn from the same subject over a longitudinal period.

As in the simulation results described previously, regardless of the size of the random effect of the subject, the two proposed methods in this paper show high coverage probabilities, which ensure the legitimacy of using them in solving practice problems. On the contrary, the current existing method gives low coverage probabilities when the random effect caused by the subject is high and non-negligible under most simulation settings. Moreover, when the challenge status of all the observations from the same subject does not change, the coverage probability of the traditional bootstrap method will be further reduced, as demonstrated in simulation study 2. However, it should be noted that when the random effect of the subject is negligible, the traditional bootstrap method is also legitimate (coverage probability approximated to 95%) and shows higher precision compared with the cluster and the hierarchical bootstrap methods.

The hierarchical bootstrap method outperforms the cluster bootstrap method and the traditional bootstrap method in terms of coverage probability; however, it gives the widest 95% CI width of AUC compared with the cluster bootstrap method and the traditional bootstrap method, which makes it less precise than the other two bootstrap methods. Also, simulation study 2 showed that the cluster bootstrap and hierarchical bootstrap methods gave similar results when the subject-level challenge status was fixed and the subject’s random effect was high. Therefore, for convenience and time efficiency in implementation, the cluster bootstrap method might be preferable to the hierarchical bootstrap method as it is legitimate and precise.

The proposed hierarchical bootstrap method was articulated for two-level cases where the total variance came from subjects and observations. This hierarchical bootstrap can be applied sequentially to each level of the hierarchical structure if there are more sources of variance. For example, multiple trials can be performed in real-life animal experiments to solve the same research question. Data collected from different trials are usually combined and analyzed as one complete study. Therefore, a trial-level random effect can emerge if each trial’s laboratory personnel, operational procedures, experimental animals, and seasons vary greatly. In this case, the hierarchical bootstrap can be performed first on different trials, then on subjects, and finally on the observations. The procedures are: (1) select trials by simple random sampling with replacement from the trials in the study; (2) select subjects within the selected trials by simple random sampling with replacement; and (3) select observations within the selected subjects by simple random sampling with replacement. In addition, the hierarchical bootstrap can be applied to multi-level data with the same principle. For example, we can consider different blocking factors, such as pen, barn, house, and sampling season, as different levels.

Given the results from our studies, we saw that the random effect of the subject is inevitable and subsequently causes issues for ROC analysis with the traditional bootstrap method. Under ideal experimental conditions, each subject should only contribute one sample to ensure that all samples are independent and identically distributed random variables. However, such ideal conditions are usually very difficult to achieve as the economic cost will be too high to afford. Therefore, most diagnostic test data correlate because researchers want to get enough samples from limited experimental subjects. Rather than suggesting that experimenters use more subjects to get samples, we recommended that researchers pay attention to possible sources of variance, consider the random effect of these sources, and use our proposed methods to do the ROC analysis if applicable. Under field conditions, multiple observations from the same subject are not as likely to be there as under experimental conditions. Yet our proposed methods are still applicable as naturally existing clusters, such as pens, barns, and houses, introduce random correlations to the diagnostic data. Further development of our methods is likely needed for application to field data, as the true status of disease is usually unknown in field studies.

One more thing to notice is that bootstrap datasets generated by the cluster bootstrap method and the hierarchical bootstrap method may have a different number of observations compared with the original dataset. This is because the number of observations from different subjects may vary in the experiment. It can be due to the experimental design or a miss of samples. As the cluster and hierarchical bootstrap methods will first bootstrap by subjects, there may be minor variations in the bootstrap sample size. However, this scenario should not affect the implementation of the proposed methods as long as most subjects have a similar number of observations drawn.

In this manuscript, we focused on testing the performance of these two proposed methods in calculating the estimated interval of AUC for a single diagnostic test. AUC comparison is another important application of ROC analysis, and it is again done with the traditional bootstrap in the pROC package. Similarly, as discussed in the introduction, we have the same concerns about comparing AUCs with the traditional bootstrap method if the data are correlated. Therefore, our future research will focus on applying these two proposed methods to compare the AUCs of different diagnostic tests.

## 5 Conclusion

In this paper, we proposed two nonparametric bootstrap methods, the cluster bootstrap, and the hierarchical bootstrap methods, for interval estimation of the area under the ROC curve for correlated diagnostic test data. Based on simulation studies, we concluded that the current existing method (the traditional bootstrap method) only works when the random effect of the subject is negligible. The traditional bootstrap method is not legitimate when there is a significant random effect of the subject. However, both methods proposed in this study show robustness in the presence of the random effect of the subject, with coverage probabilities close to or higher than 95%. In the analysis of real data, the cluster bootstrap method and the hierarchical bootstrap method give similar estimations of the results; however, the cluster bootstrap method gives narrower confidence intervals in comparison with the hierarchical bootstrap method, while the hierarchical bootstrap method always has a higher coverage probability among all the three methods. The two proposed methods will be helpful in analyzing correlated data in experimental diagnostic studies where multiple observations are collected from the same subject over a period of time. In this work, we evaluated a new diagnostic test’s ability to distinguish a random abnormal subject from a random normal subject, which is the traditional AUC at the population level. It should be noted that the predictive ability of a diagnostic test at the subject level (within a subject) and at the population level (between subject) are not the same (22). If the focus is to evaluate the diagnostic test within a subject, our method can be modified to simulate within subject resampling to achieve that goal.

## Data availability statement

The datasets presented in this study can be found in online repositories. The names of the repository/repositories and accession number(s) can be found in the article/supplementary material.

## Author contributions

JP: Formal analysis, Methodology, Software, Writing – original draft, Writing – review & editing. WJ: Software, Writing – review & editing. MW: Writing – review & editing. PG: Writing – review & editing. PL: Writing – review & editing. QZ: Writing – review & editing. CW: Conceptualization, Formal analysis, Funding acquisition, Methodology, Project administration, Resources, Supervision, Writing – original draft, Writing – review & editing.
